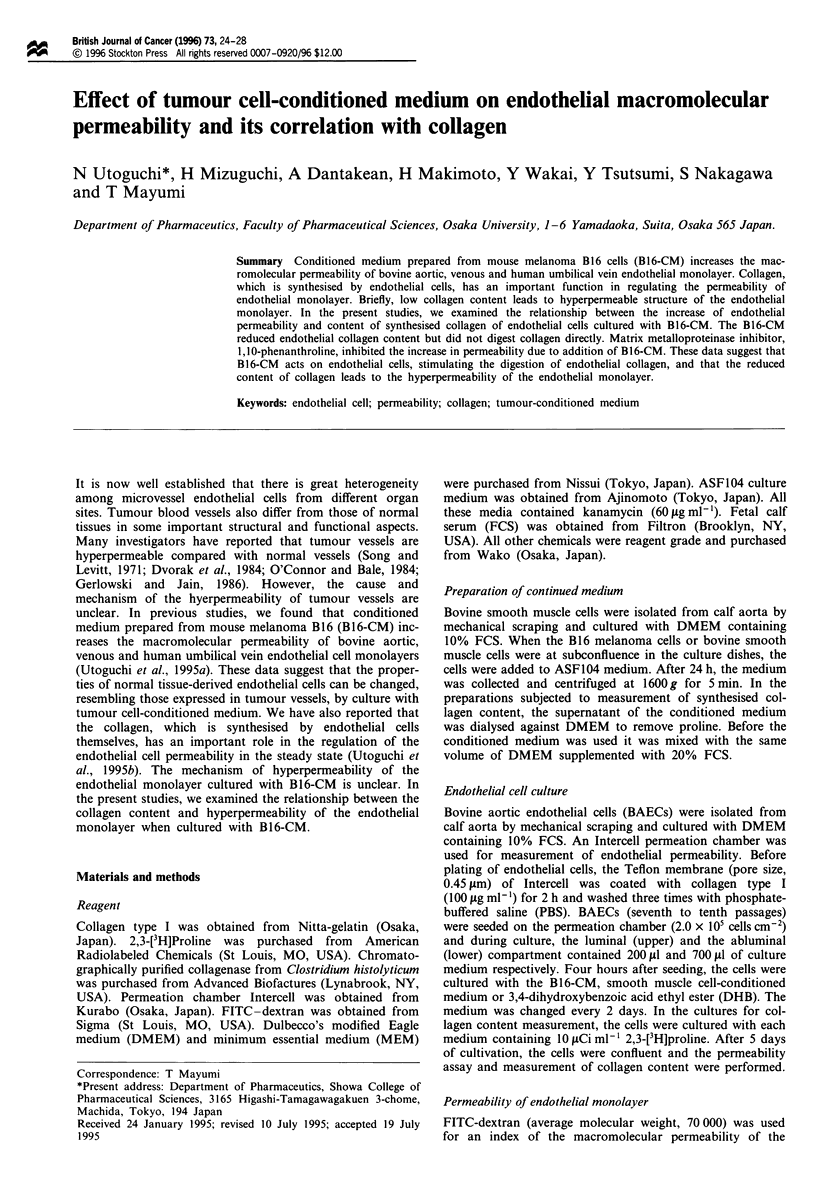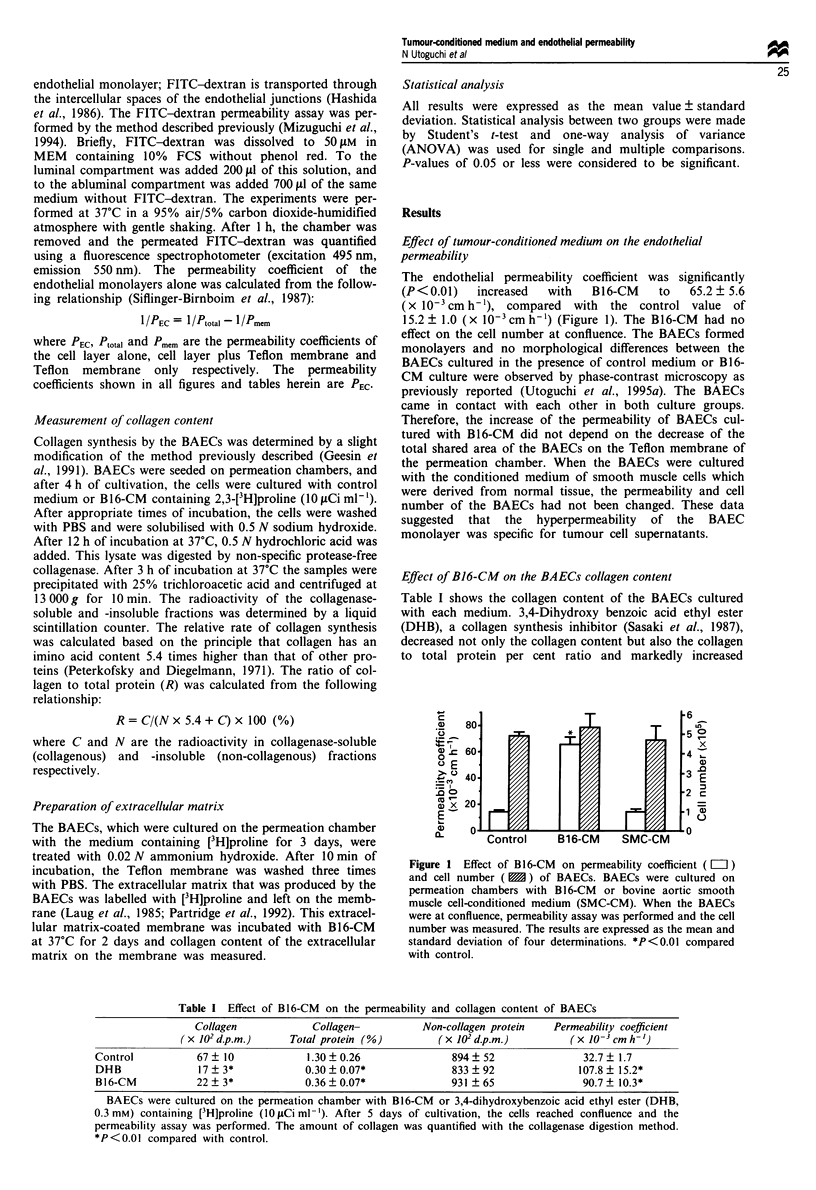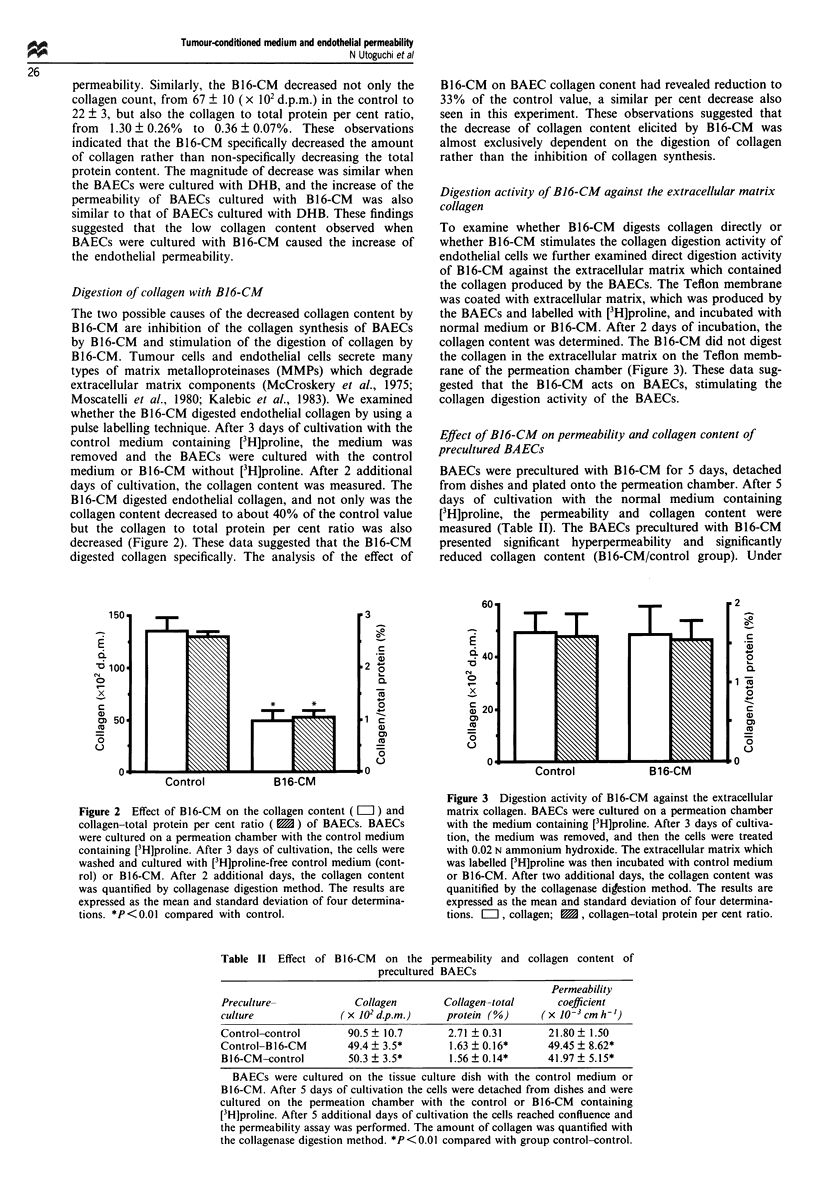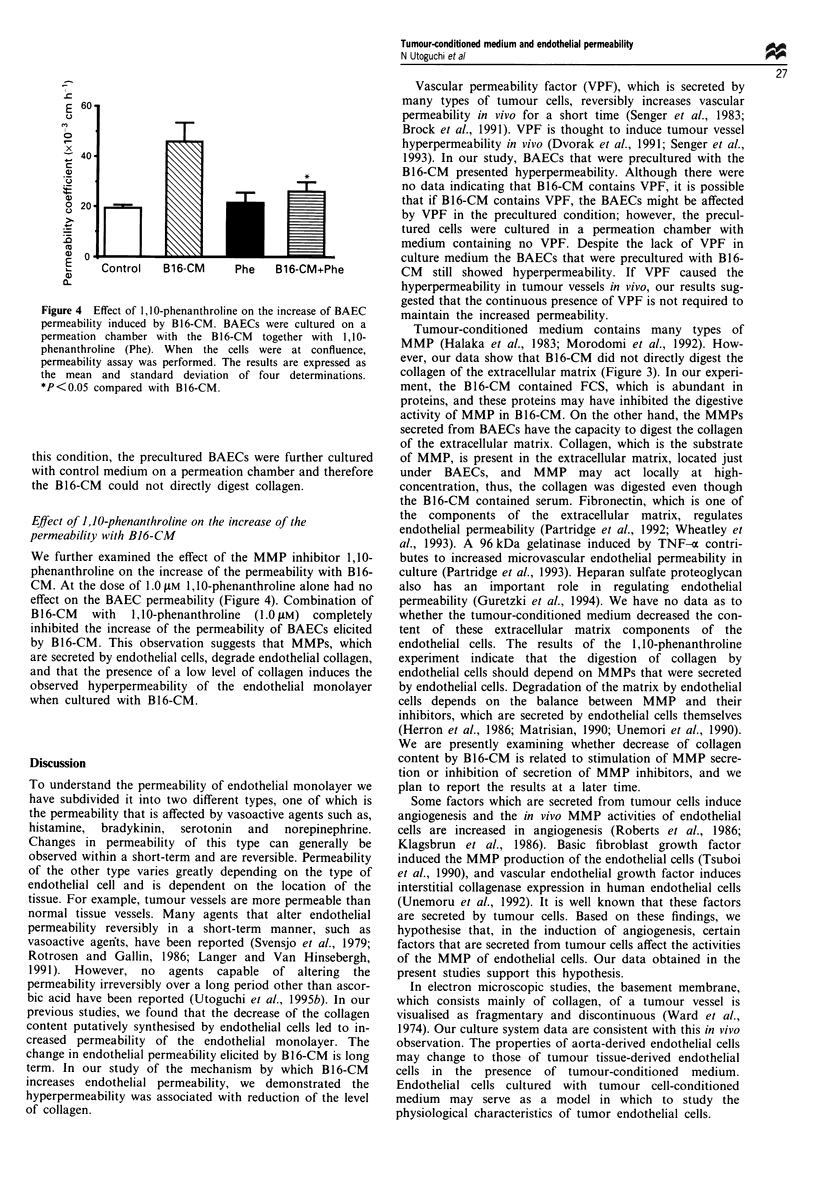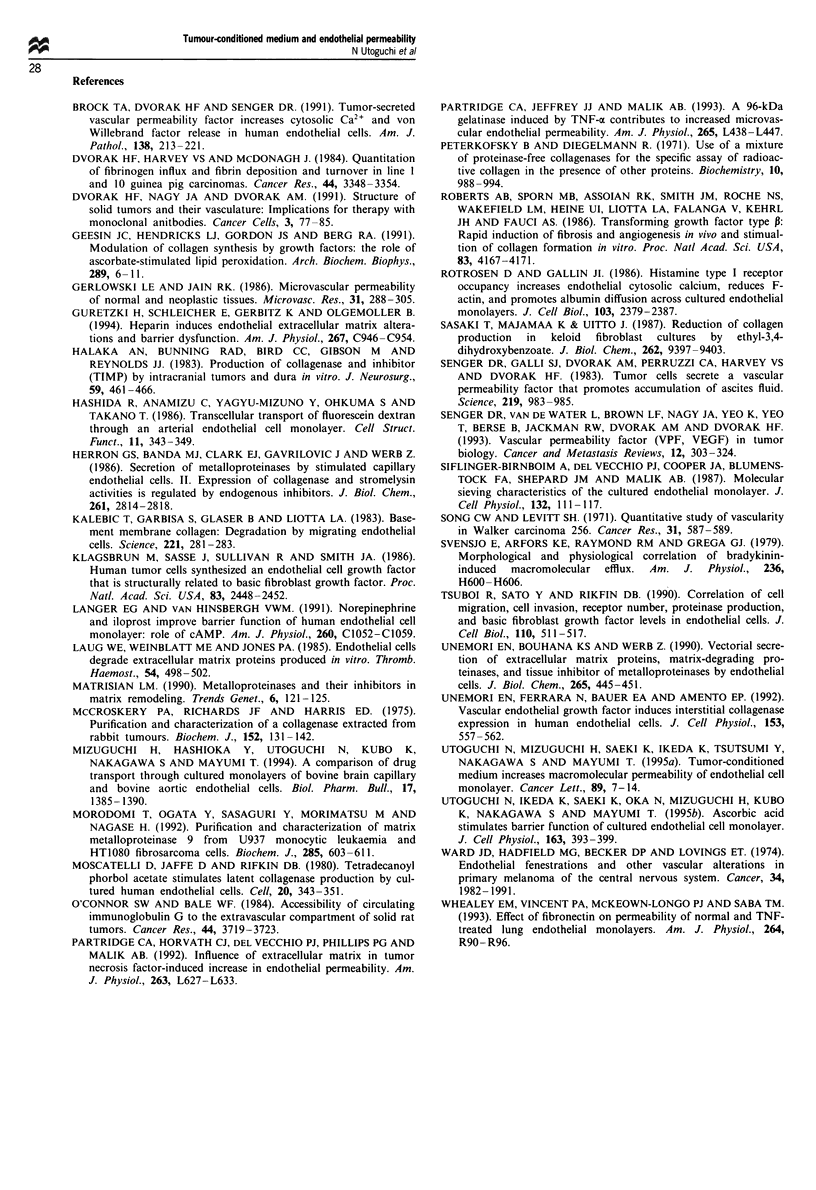# Effect of tumour cell-conditioned medium on endothelial macromolecular permeability and its correlation with collagen.

**DOI:** 10.1038/bjc.1996.5

**Published:** 1996-01

**Authors:** N. Utoguchi, H. Mizuguchi, A. Dantakean, H. Makimoto, Y. Wakai, Y. Tsutsumi, S. Nakagawa, T. Mayumi

**Affiliations:** Department of Pharmaceutics, Faculty of Pharmaceutical Sciences, Osaka University, Japan.

## Abstract

Conditioned medium prepared from mouse melanoma B16 cells (B16-CM) increases the macromolecular permeability of bovine aortic, venous and human umbilical vein endothelial monolayer. Collagen, which is synthesised by endothelial cells, has an important function in regulating the permeability of endothelial monolayer. Briefly, low collagen content leads to hyperpermeable structure of the endothelial monolayer. In the present studies, we examined the relationship between the increase of endothelial permeability and content of synthesised collagen of endothelial cells cultured with B16-CM. The B16-CM reduced endothelial collagen content but did not digest collagen directly. Matrix metalloproteinase inhibitor, 1,10-phenanthroline, inhibited the increase in permeability due to addition of B16-CM. These data suggest that B16-CM acts on endothelial cells, stimulating the digestion of endothelial collagen, and that the reduced content of collagen leads to the hyperpermeability of the endothelial monolayer.


British Journal of Cancer (1996) 73, 24-28
© 1996 Stockton Press All rights reserved 0007-0920/96 $12.00

### Effect of tumour cell-conditioned medium on endothelial macromolecular permeability and its correlation with collagen

N Utoguchi*, H Mizuguchi, A Dantakean, H Makimoto, Y Wakai, Y Tsutsumi, S Nakagawa and T Mayumi

*Department of Pharmaceutics, Faculty of Pharmaceutical Sciences, Osaka University, 1-6 Yamadaoka, Suita, Osaka 565 Japan.*

**Summary** Conditioned medium prepared from mouse melanoma B16 cells (B16-CM) increases the macromolecular permeability of bovine aortic, venous and human umbilical vein endothelial monolayer. Collagen, which is synthesised by endothelial cells, has an important function in regulating the permeability of endothelial monolayer. Briefly, low collagen content leads to hyperpermeable structure of the endothelial monolayer. In the present studies, we examined the relationship between the increase of endothelial permeability and content of synthesised collagen of endothelial cells cultured with B16-CM. The B16-CM reduced endothelial collagen content but did not digest collagen directly. Matrix metalloproteinase inhibitor, 1,10-phenanthroline, inhibited the increase in permeability due to addition of B16-CM. These data suggest that B16-CM acts on endothelial cells, stimulating the digestion of endothelial collagen, and that the reduced content of collagen leads to the hyperpermeability of the endothelial monolayer.

**Keywords:** endothelial cell; permeability; collagen; tumour-conditioned medium

It is now well established that there is great heterogeneity among microvessel endothelial cells from different organ sites. Tumour blood vessels also differ from those of normal tissues in some important structural and functional aspects. Many investigators have reported that tumour vessels are hyperpermeable compared with normal vessels (Song and Levitt, 1971; Dvorak *et al.*, 1984; O'Connor and Bale, 1984; Gerlowski and Jain, 1986). However, the cause and mechanism of the hyerpermeability of tumour vessels are unclear. In previous studies, we found that conditioned medium prepared from mouse melanoma B16 (B16-CM) increases the macromolecular permeability of bovine aortic, venous and human umbilical vein endothelial cell monolayers (Utoguchi *et al.*, 1995*a*). These data suggest that the properties of normal tissue-derived endothelial cells can be changed, resembling those expressed in tumour vessels, by culture with tumour cell-conditioned medium. We have also reported that the collagen, which is synthesised by endothelial cells themselves, has an important role in the regulation of the endothelial cell permeability in the steady state (Utoguchi *et al.*, 1995*b*). The mechanism of hyperpermeability of the endothelial monolayer cultured with B16-CM is unclear. In the present studies, we examined the relationship between the collagen content and hyperpermeability of the endothelial monolayer when cultured with B16-CM.

### Materials and methods

### *Reagent*

Collagen type I was obtained from Nitta-gelatin (Osaka, Japan). 2,3-[$^3$H]Proline was purchased from American Radiolabeled Chemicals (St Louis, MO, USA). Chromatographically purified collagenase from *Clostridium histolyticum* was purchased from Advanced Biofactures (Lynabrook, NY, USA). Permeation chamber Intercell was obtained from Kurabo (Osaka, Japan). FITC-dextran was obtained from Sigma (St Louis, MO, USA). Dulbecco's modified Eagle medium (DMEM) and minimum essential medium (MEM) were purchased from Nissui (Tokyo, Japan). ASF104 culture medium was obtained from Ajinomoto (Tokyo, Japan). All these media contained kanamycin (60 $\mu$g ml$^{-1}$). Fetal calf serum (FCS) was obtained from Filtron (Brooklyn, NY, USA). All other chemicals were reagent grade and purchased from Wako (Osaka, Japan).

### *Preparation of continued medium*

Bovine smooth muscle cells were isolated from calf aorta by mechanical scraping and cultured with DMEM containing 10% FCS. When the B16 melanoma cells or bovine smooth muscle cells were at subconfluence in the culture dishes, the cells were added to ASF104 medium. After 24 h, the medium was collected and centrifuged at 1600 *g* for 5 min. In the preparations subjected to measurement of synthesised collagen content, the supernatant of the conditioned medium was dialysed against DMEM to remove proline. Before the conditioned medium was used it was mixed with the same volume of DMEM supplemented with 20% FCS.

### *Endothelial cell culture*

Bovine aortic endothelial cells (BAECs) were isolated from calf aorta by mechanical scraping and cultured with DMEM containing 10% FCS. An Intercell permeation chamber was used for measurement of endothelial permeability. Before plating of endothelial cells, the Teflon membrane (pore size, 0.45 $\mu$m) of Intercell was coated with collagen type I (100 $\mu$g ml$^{-1}$) for 2 h and washed three times with phosphate-buffered saline (PBS). BAECs (seventh to tenth passages) were seeded on the permeation chamber (2.0 × 10$^5$ cells cm$^{-2}$) and during culture, the luminal (upper) and the abluminal (lower) compartment contained 200 $\mu$l and 700 $\mu$l of culture medium respectively. Four hours after seeding, the cells were cultured with the B16-CM, smooth muscle cell-conditioned medium or 3,4-dihydroxybenzoic acid ethyl ester (DHB). The medium was changed every 2 days. In the cultures for collagen content measurement, the cells were cultured with each medium containing 10 $\mu$Ci ml$^{-1}$ 2,3-[$^3$H]proline. After 5 days of cultivation, the cells were confluent and the permeability assay and measurement of collagen content were performed.

### *Permeability of endothelial monolayer*

FITC-dextran (average molecular weight, 70 000) was used for an index of the macromolecular permeability of the

Correspondence: T Mayumi
*Present address: Department of Pharmaceutics, Showa College of Pharmaceutical Sciences, 3165 Higashi-Tamagawagakuen 3-chome, Machida, Tokyo, 194 Japan
Received 24 January 1995; revised 10 July 1995; accepted 19 July 1995

endothelial monolayer; FITC–dextran is transported through the intercellular spaces of the endothelial junctions (Hashida *et al.*, 1986). The FITC–dextran permeability assay was performed by the method described previously (Mizuguchi *et al.*, 1994). Briefly, FITC–dextran was dissolved to 50 μM in MEM containing 10% FCS without phenol red. To the luminal compartment was added 200 μl of this solution, and to the abluminal compartment was added 700 μl of the same medium without FITC–dextran. The experiments were performed at 37°C in a 95% air/5% carbon dioxide-humidified atmosphere with gentle shaking. After 1 h, the chamber was removed and the permeated FITC–dextran was quantified using a fluorescence spectrophotometer (excitation 495 nm, emission 550 nm). The permeability coefficient of the endothelial monolayers alone was calculated from the following relationship (Siflinger-Birnboim *et al.*, 1987):

$$1/P_{EC} = 1/P_{total} - 1/P_{mem}$$

where $P_{EC}$, $P_{total}$ and $P_{mem}$ are the permeability coefficients of the cell layer alone, cell layer plus Teflon membrane and Teflon membrane only respectively. The permeability coefficients shown in all figures and tables herein are $P_{EC}$.

### *Measurement of collagen content*

Collagen synthesis by the BAECs was determined by a slight modification of the method previously described (Geesin *et al.*, 1991). BAECs were seeded on permeation chambers, and after 4 h of cultivation, the cells were cultured with control medium or B16-CM containing 2,3-[$^3$H]proline (10 μCi ml$^{-1}$). After appropriate times of incubation, the cells were washed with PBS and were solubilised with 0.5 *N* sodium hydroxide. After 12 h of incubation at 37°C, 0.5 *N* hydrochloric acid was added. This lysate was digested by non-specific protease-free collagenase. After 3 h of incubation at 37°C the samples were precipitated with 25% trichloroacetic acid and centrifuged at 13 000 *g* for 10 min. The radioactivity of the collagenase-soluble and -insoluble fractions was determined by a liquid scintillation counter. The relative rate of collagen synthesis was calculated based on the principle that collagen has an imino acid content 5.4 times higher than that of other proteins (Peterkofsky and Diegelmann, 1971). The ratio of collagen to total protein (*R*) was calculated from the following relationship:

$$R = C/(N \times 5.4 + C) \times 100\ (\%)$$

where *C* and *N* are the radioactivity in collagenase-soluble (collagenous) and -insoluble (non-collagenous) fractions respectively.

### *Preparation of extracellular matrix*

The BAECs, which were cultured on the permeation chamber with the medium containing [$^3$H]proline for 3 days, were treated with 0.02 *N* ammonium hydroxide. After 10 min of incubation, the Teflon membrane was washed three times with PBS. The extracellular matrix that was produced by the BAECs was labelled with [$^3$H]proline and left on the membrane (Laug *et al.*, 1985; Partridge *et al.*, 1992). This extracellular matrix-coated membrane was incubated with B16-CM at 37°C for 2 days and collagen content of the extracellular matrix on the membrane was measured.

### *Statistical analysis*

All results were expressed as the mean value ± standard deviation. Statistical analysis between two groups were made by Student's *t*-test and one-way analysis of variance (ANOVA) was used for single and multiple comparisons. *P*-values of 0.05 or less were considered to be significant.

### Results

### *Effect of tumour-conditioned medium on the endothelial permeability*

The endothelial permeability coefficient was significantly ($P<0.01$) increased with B16-CM to 65.2 ± 5.6 (× 10$^{-3}$ cm h$^{-1}$), compared with the control value of 15.2 ± 1.0 (× 10$^{-3}$ cm h$^{-1}$) (Figure 1). The B16-CM had no effect on the cell number at confluence. The BAECs formed monolayers and no morphological differences between the BAECs cultured in the presence of control medium or B16-CM culture were observed by phase-contrast microscopy as previously reported (Utoguchi *et al.*, 1995*a*). The BAECs came in contact with each other in both culture groups. Therefore, the increase of the permeability of BAECs cultured with B16-CM did not depend on the decrease of the total shared area of the BAECs on the Teflon membrane of the permeation chamber. When the BAECs were cultured with the conditioned medium of smooth muscle cells which were derived from normal tissue, the permeability and cell number of the BAECs had not been changed. These data suggested that the hyperpermeability of the BAEC monolayer was specific for tumour cell supernatants.

### *Effect of B16-CM on the BAECs collagen content*

Table I shows the collagen content of the BAECs cultured with each medium. 3,4-Dihydroxy benzoic acid ethyl ester (DHB), a collagen synthesis inhibitor (Sasaki *et al.*, 1987), decreased not only the collagen content but also the collagen to total protein per cent ratio and markedly increased

**Figure 1** Effect of B16-CM on permeability coefficient (□) and cell number (▨) of BAECs. BAECs were cultured on permeation chambers with B16-CM or bovine aortic smooth muscle cell-conditioned medium (SMC-CM). When the BAECs were at confluence, permeability assay was performed and the cell number was measured. The results are expressed as the mean and standard deviation of four determinations. *$P<0.01$ compared with control.

**Table I** Effect of B16-CM on the permeability and collagen content of BAECs

| | *Collagen* (× 10$^2$ *d.p.m.*) | *Collagen–Total protein* (%) | *Non-collagen protein* (× 10$^2$ *d.p.m.*) | *Permeability coefficient* (× 10$^{-3}$ *cm h$^{-1}$*) |
|---|---|---|---|---|
| Control | 67 ± 10 | 1.30 ± 0.26 | 894 ± 52 | 32.7 ± 1.7 |
| DHB | 17 ± 3* | 0.30 ± 0.07* | 833 ± 92 | 107.8 ± 15.2* |
| B16-CM | 22 ± 3* | 0.36 ± 0.07* | 931 ± 65 | 90.7 ± 10.3* |

BAECs were cultured on the permeation chamber with B16-CM or 3,4-dihydroxybenzoic acid ethyl ester (DHB, 0.3 mM) containing [$^3$H]proline (10 μCi ml$^{-1}$). After 5 days of cultivation, the cells reached confluence and the permeability assay was performed. The amount of collagen was quantified with the collagenase digestion method. *$P<0.01$ compared with control.

permeability. Similarly, the B16-CM decreased not only the collagen count, from 67 ± 10 ( × $10^2$ d.p.m.) in the control to 22 ± 3, but also the collagen to total protein per cent ratio, from 1.30 ± 0.26% to 0.36 ± 0.07%. These observations indicated that the B16-CM specifically decreased the amount of collagen rather than non-specifically decreasing the total protein content. The magnitude of decrease was similar when the BAECs were cultured with DHB, and the increase of the permeability of BAECs cultured with B16-CM was also similar to that of BAECs cultured with DHB. These findings suggested that the low collagen content observed when BAECs were cultured with B16-CM caused the increase of the endothelial permeability.

*Digestion of collagen with B16-CM*

The two possible causes of the decreased collagen content by B16-CM are inhibition of the collagen synthesis of BAECs by B16-CM and stimulation of the digestion of collagen by B16-CM. Tumour cells and endothelial cells secrete many types of matrix metalloproteinases (MMPs) which degrade extracellular matrix components (McCroskery *et al.*, 1975; Moscatelli *et al.*, 1980; Kalebic *et al.*, 1983). We examined whether the B16-CM digested endothelial collagen by using a pulse labelling technique. After 3 days of cultivation with the control medium containing [$^3$H]proline, the medium was removed and the BAECs were cultured with the control medium or B16-CM without [$^3$H]proline. After 2 additional days of cultivation, the collagen content was measured. The B16-CM digested endothelial collagen, and not only was the collagen content decreased to about 40% of the control value but the collagen to total protein per cent ratio was also decreased (Figure 2). These data suggested that the B16-CM digested collagen specifically. The analysis of the effect of B16-CM on BAEC collagen conent had revealed reduction to 33% of the control value, a similar per cent decrease also seen in this experiment. These observations suggested that the decrease of collagen content elicited by B16-CM was almost exclusively dependent on the digestion of collagen rather than the inhibition of collagen synthesis.

*Digestion activity of B16-CM against the extracellular matrix collagen*

To examine whether B16-CM digests collagen directly or whether B16-CM stimulates the collagen digestion activity of endothelial cells we further examined direct digestion activity of B16-CM against the extracellular matrix which contained the collagen produced by the BAECs. The Teflon membrane was coated with extracellular matrix, which was produced by the BAECs and labelled with [$^3$H]proline, and incubated with normal medium or B16-CM. After 2 days of incubation, the collagen content was determined. The B16-CM did not digest the collagen in the extracellular matrix on the Teflon membrane of the permeation chamber (Figure 3). These data suggested that the B16-CM acts on BAECs, stimulating the collagen digestion activity of the BAECs.

*Effect of B16-CM on permeability and collagen content of precultured BAECs*

BAECs were precultured with B16-CM for 5 days, detached from dishes and plated onto the permeation chamber. After 5 days of cultivation with the normal medium containing [$^3$H]proline, the permeability and collagen content were measured (Table II). The BAECs precultured with B16-CM presented significant hyperpermeability and significantly reduced collagen content (B16-CM/control group). Under

**Figure 2** Effect of B16-CM on the collagen content ( □ ) and collagen–total protein per cent ratio ( ▨ ) of BAECs. BAECs were cultured on a permeation chamber with the control medium containing [$^3$H]proline. After 3 days of cultivation, the cells were washed and cultured with [$^3$H]proline-free control medium (control) or B16-CM. After 2 additional days, the collagen content was quantified by collagenase digestion method. The results are expressed as the mean and standard deviation of four determinations. *$P<0.01$ compared with control.

**Figure 3** Digestion activity of B16-CM against the extracellular matrix collagen. BAECs were cultured on a permeation chamber with the medium containing [$^3$H]proline. After 3 days of cultivation, the medium was removed, and then the cells were treated with 0.02 N ammonium hydroxide. The extracellular matrix which was labelled [$^3$H]proline was then incubated with control medium or B16-CM. After two additional days, the collagen content was quanitified by the collagenase digestion method. The results are expressed as the mean and standard deviation of four determinations. □ , collagen; ▨ , collagen–total protein per cent ratio.

**Table II** Effect of B16-CM on the permeability and collagen content of precultured BAECs

| *Preculture–culture* | *Collagen ( × $10^2$ d.p.m.)* | *Collagen–total protein (%)* | *Permeability coefficient ( × $10^{-3}$ cm $h^{-1}$)* |
|---|---|---|---|
| Control–control | 90.5 ± 10.7 | 2.71 ± 0.31 | 21.80 ± 1.50 |
| Control–B16-CM | 49.4 ± 3.5* | 1.63 ± 0.16* | 49.45 ± 8.62* |
| B16-CM–control | 50.3 ± 3.5* | 1.56 ± 0.14* | 41.97 ± 5.15* |

BAECs were cultured on the tissue culture dish with the control medium or B16-CM. After 5 days of cultivation the cells were detached from dishes and were cultured on the permeation chamber with the control or B16-CM containing [$^3$H]proline. After 5 additional days of cultivation the cells reached confluence and the permeability assay was performed. The amount of collagen was quantified with the collagenase digestion method. *$P<0.01$ compared with group control–control.

**Figure 4** Effect of 1,10-phenanthroline on the increase of BAEC permeability induced by B16-CM. BAECs were cultured on a permeation chamber with the B16-CM together with 1,10-phenanthroline (Phe). When the cells were at confluence, permeability assay was performed. The results are expressed as the mean and standard deviation of four determinations. *$P<0.05$ compared with B16-CM.

this condition, the precultured BAECs were further cultured with control medium on a permeation chamber and therefore the B16-CM could not directly digest collagen.

*Effect of 1,10-phenanthroline on the increase of the permeability with B16-CM*

We further examined the effect of the MMP inhibitor 1,10-phenanthroline on the increase of the permeability with B16-CM. At the dose of 1.0 μM 1,10-phenanthroline alone had no effect on the BAEC permeability (Figure 4). Combination of B16-CM with 1,10-phenanthroline (1.0 μM) completely inhibited the increase of the permeability of BAECs elicited by B16-CM. This observation suggests that MMPs, which are secreted by endothelial cells, degrade endothelial collagen, and that the presence of a low level of collagen induces the observed hyperpermeability of the endothelial monolayer when cultured with B16-CM.

### Discussion

To understand the permeability of endothelial monolayer we have subdivided it into two different types, one of which is the permeability that is affected by vasoactive agents such as, histamine, bradykinin, serotonin and norepinephrine. Changes in permeability of this type can generally be observed within a short-term and are reversible. Permeability of the other type varies greatly depending on the type of endothelial cell and is dependent on the location of the tissue. For example, tumour vessels are more permeable than normal tissue vessels. Many agents that alter endothelial permeability reversibly in a short-term manner, such as vasoactive agents, have been reported (Svensjo *et al.*, 1979; Rotrosen and Gallin, 1986; Langer and Van Hinsebergh, 1991). However, no agents capable of altering the permeability irreversibly over a long period other than ascorbic acid have been reported (Utoguchi *et al.*, 1995*b*). In our previous studies, we found that the decrease of the collagen content putatively synthesised by endothelial cells led to increased permeability of the endothelial monolayer. The change in endothelial permeability elicited by B16-CM is long term. In our study of the mechanism by which B16-CM increases endothelial permeability, we demonstrated the hyperpermeability was associated with reduction of the level of collagen.

Vascular permeability factor (VPF), which is secreted by many types of tumour cells, reversibly increases vascular permeability *in vivo* for a short time (Senger *et al.*, 1983; Brock *et al.*, 1991). VPF is thought to induce tumour vessel hyperpermeability *in vivo* (Dvorak *et al.*, 1991; Senger *et al.*, 1993). In our study, BAECs that were precultured with the B16-CM presented hyperpermeability. Although there were no data indicating that B16-CM contains VPF, it is possible that if B16-CM contains VPF, the BAECs might be affected by VPF in the precultured condition; however, the precultured cells were cultured in a permeation chamber with medium containing no VPF. Despite the lack of VPF in culture medium the BAECs that were precultured with B16-CM still showed hyperpermeability. If VPF caused the hyperpermeability in tumour vessels *in vivo*, our results suggested that the continuous presence of VPF is not required to maintain the increased permeability.

Tumour-conditioned medium contains many types of MMP (Halaka *et al.*, 1983; Morodomi *et al.*, 1992). However, our data show that B16-CM did not directly digest the collagen of the extracellular matrix (Figure 3). In our experiment, the B16-CM contained FCS, which is abundant in proteins, and these proteins may have inhibited the digestive activity of MMP in B16-CM. On the other hand, the MMPs secreted from BAECs have the capacity to digest the collagen of the extracellular matrix. Collagen, which is the substrate of MMP, is present in the extracellular matrix, located just under BAECs, and MMP may act locally at high-concentration, thus, the collagen was digested even though the B16-CM contained serum. Fibronectin, which is one of the components of the extracellular matrix, regulates endothelial permeability (Partridge *et al.*, 1992; Wheatley *et al.*, 1993). A 96 kDa gelatinase induced by TNF-α contributes to increased microvascular endothelial permeability in culture (Partridge *et al.*, 1993). Heparan sulfate proteoglycan also has an important role in regulating endothelial permeability (Guretzki *et al.*, 1994). We have no data as to whether the tumour-conditioned medium decreased the content of these extracellular matrix components of the endothelial cells. The results of the 1,10-phenanthroline experiment indicate that the digestion of collagen by endothelial cells should depend on MMPs that were secreted by endothelial cells. Degradation of the matrix by endothelial cells depends on the balance between MMP and their inhibitors, which are secreted by endothelial cells themselves (Herron *et al.*, 1986; Matrisian, 1990; Unemori *et al.*, 1990). We are presently examining whether decrease of collagen content by B16-CM is related to stimulation of MMP secretion or inhibition of secretion of MMP inhibitors, and we plan to report the results at a later time.

Some factors which are secreted from tumour cells induce angiogenesis and the *in vivo* MMP activities of endothelial cells are increased in angiogenesis (Roberts *et al.*, 1986; Klagsbrun *et al.*, 1986). Basic fibroblast growth factor induced the MMP production of the endothelial cells (Tsuboi *et al.*, 1990), and vascular endothelial growth factor induces interstitial collagenase expression in human endothelial cells (Unemoru *et al.*, 1992). It is well known that these factors are secreted by tumour cells. Based on these findings, we hypothesise that, in the induction of angiogenesis, certain factors that are secreted from tumour cells affect the activities of the MMP of endothelial cells. Our data obtained in the present studies support this hypothesis.

In electron microscopic studies, the basement membrane, which consists mainly of collagen, of a tumour vessel is visualised as fragmentary and discontinuous (Ward *et al.*, 1974). Our culture system data are consistent with this *in vivo* observation. The properties of aorta-derived endothelial cells may change to those of tumour tissue-derived endothelial cells in the presence of tumour-conditioned medium. Endothelial cells cultured with tumour cell-conditioned medium may serve as a model in which to study the physiological characteristics of tumor endothelial cells.

### References

BROCK TA, DVORAK HF AND SENGER DR. (1991). Tumor-secreted vascular permeability factor increases cytosolic $Ca^{2+}$ and von Willebrand factor release in human endothelial cells. *Am. J. Pathol.*, **138**, 213–221.

DVORAK HF, HARVEY VS AND MCDONAGH J. (1984). Quantitation of fibrinogen influx and fibrin deposition and turnover in line 1 and 10 guinea pig carcinomas. *Cancer Res.*, **44**, 3348–3354.

DVORAK HF, NAGY JA AND DVORAK AM. (1991). Structure of solid tumors and their vasculature: Implications for therapy with monoclonal anitbodies. *Cancer Cells*, **3**, 77–85.

GEESIN JC, HENDRICKS LJ, GORDON JS AND BERG RA. (1991). Modulation of collagen synthesis by growth factors: the role of ascorbate-stimulated lipid peroxidation. *Arch. Biochem. Biophys.*, **289**, 6–11.

GERLOWSKI LE AND JAIN RK. (1986). Microvascular permeability of normal and neoplastic tissues. *Microvasc. Res.*, **31**, 288–305.

GURETZKI H, SCHLEICHER E, GERBITZ K AND OLGEMOLLER B. (1994). Heparin induces endothelial extracellular matrix alterations and barrier dysfunction. *Am. J. Physiol.*, **267**, C946–C954.

HALAKA AN, BUNNING RAD, BIRD CC, GIBSON M AND REYNOLDS JJ. (1983). Production of collagenase and inhibitor (TIMP) by intracranial tumors and dura *in vitro*. *J. Neurosurg.*, **59**, 461–466.

HASHIDA R, ANAMIZU C, YAGYU-MIZUNO Y, OHKUMA S AND TAKANO T. (1986). Transcellular transport of fluorescein dextran through an arterial endothelial cell monolayer. *Cell Struct. Funct.*, **11**, 343–349.

HERRON GS, BANDA MJ, CLARK EJ, GAVRILOVIC J AND WERB Z. (1986). Secretion of metalloproteinases by stimulated capillary endothelial cells. II. Expression of collagenase and stromelysin activities is regulated by endogenous inhibitors. *J. Biol. Chem.*, **261**, 2814–2818.

KALEBIC T, GARBISA S, GLASER B AND LIOTTA LA. (1983). Basement membrane collagen: Degradation by migrating endothelial cells. *Science*, **221**, 281–283.

KLAGSBRUN M, SASSE J, SULLIVAN R AND SMITH JA. (1986). Human tumor cells synthesized an endothelial cell growth factor that is structurally related to basic fibroblast growth factor. *Proc. Natl. Acad. Sci. USA*, **83**, 2448–2452.

LANGER EG AND VAN HINSBERGH VWM. (1991). Norepinephrine and iloprost improve barrier function of human endothelial cell monolayer: role of cAMP. *Am. J. Physiol.*, **260**, C1052–C1059.

LAUG WE, WEINBLATT ME AND JONES PA. (1985). Endothelial cells degrade extracellular matrix proteins produced *in vitro*. *Thromb. Haemost.*, **54**, 498–502.

MATRISIAN LM. (1990). Metalloproteinases and their inhibitors in matrix remodeling. *Trends Genet.*, **6**, 121–125.

MCCROSKERY PA, RICHARDS JF AND HARRIS ED. (1975). Purification and characterization of a collagenase extracted from rabbit tumours. *Biochem. J.*, **152**, 131–142.

MIZUGUCHI H, HASHIOKA Y, UTOGUCHI N, KUBO K, NAKAGAWA S AND MAYUMI T. (1994). A comparison of drug transport through cultured monolayers of bovine brain capillary and bovine aortic endothelial cells. *Biol. Pharm. Bull.*, **17**, 1385–1390.

MORODOMI T, OGATA Y, SASAGURI Y, MORIMATSU M AND NAGASE H. (1992). Purification and characterization of matrix metalloproteinase 9 from U937 monocytic leukaemia and HT1080 fibrosarcoma cells. *Biochem. J.*, **285**, 603–611.

MOSCATELLI D, JAFFE D AND RIFKIN DB. (1980). Tetradecanoyl phorbol acetate stimulates latent collagenase production by cultured human endothelial cells. *Cell*, **20**, 343–351.

O'CONNOR SW AND BALE WF. (1984). Accessibility of circulating immunoglobulin G to the extravascular compartment of solid rat tumors. *Cancer Res.*, **44**, 3719–3723.

PARTRIDGE CA, HORVATH CJ, DEL VECCHIO PJ, PHILLIPS PG AND MALIK AB. (1992). Influence of extracellular matrix in tumor necrosis factor-induced increase in endothelial permeability. *Am. J. Physiol.*, **263**, L627–L633.

PARTRIDGE CA, JEFFREY JJ AND MALIK AB. (1993). A 96-kDa gelatinase induced by TNF-α contributes to increased microvascular endothelial permeability. *Am. J. Physiol.*, **265**, L438–L447.

PETERKOFSKY B AND DIEGELMANN R. (1971). Use of a mixture of proteinase-free collagenases for the specific assay of radioactive collagen in the presence of other proteins. *Biochemistry*, **10**, 988–994.

ROBERTS AB, SPORN MB, ASSOIAN RK, SMITH JM, ROCHE NS, WAKEFIELD LM, HEINE UI, LIOTTA LA, FALANGA V, KEHRL JH AND FAUCI AS. (1986). Transforming growth factor type β: Rapid induction of fibrosis and angiogenesis *in vivo* and stimualtion of collagen formation *in vitro*. *Proc. Natl Acad. Sci. USA*, **83**, 4167–4171.

ROTROSEN D AND GALLIN JI. (1986). Histamine type I receptor occupancy increases endothelial cytosolic calcium, reduces F-actin, and promotes albumin diffusion across cultured endothelial monolayers. *J. Cell Biol.*, **103**, 2379–2387.

SASAKI T, MAJAMAA K & UITTO J. (1987). Reduction of collagen production in keloid fibroblast cultures by ethyl-3,4-dihydroxybenzoate. *J. Biol. Chem.*, **262**, 9397–9403.

SENGER DR, GALLI SJ, DVORAK AM, PERRUZZI CA, HARVEY VS AND DVORAK HF. (1983). Tumor cells secrete a vascular permeability factor that promotes accumulation of ascites fluid. *Science*, **219**, 983–985.

SENGER DR, VAN DE WATER L, BROWN LF, NAGY JA, YEO K, YEO T, BERSE B, JACKMAN RW, DVORAK AM AND DVORAK HF. (1993). Vascular permeability factor (VPF, VEGF) in tumor biology. *Cancer and Metastasis Reviews*, **12**, 303–324.

SIFLINGER-BIRNBOIM A, DEL VECCHIO PJ, COOPER JA, BLUMENSTOCK FA, SHEPARD JM AND MALIK AB. (1987). Molecular sieving characteristics of the cultured endothelial monolayer. *J. Cell Physiol.*, **132**, 111–117.

SONG CW AND LEVITT SH. (1971). Quantitative study of vascularity in Walker carcinoma 256. *Cancer Res.*, **31**, 587–589.

SVENSJO E, ARFORS KE, RAYMOND RM AND GREGA GJ. (1979). Morphological and physiological correlation of bradykinin-induced macromolecular efflux. *Am. J. Physiol.*, **236**, H600–H606.

TSUBOI R, SATO Y AND RIKFIN DB. (1990). Correlation of cell migration, cell invasion, receptor number, proteinase production, and basic fibroblast growth factor levels in endothelial cells. *J. Cell Biol.*, **110**, 511–517.

UNEMORI EN, BOUHANA KS AND WERB Z. (1990). Vectorial secretion of extracellular matrix proteins, matrix-degrading proteinases, and tissue inhibitor of metalloproteinases by endothelial cells. *J. Biol. Chem.*, **265**, 445–451.

UNEMORI EN, FERRARA N, BAUER EA AND AMENTO EP. (1992). Vascular endothelial growth factor induces interstitial collagenase expression in human endothelial cells. *J. Cell Physiol.*, **153**, 557–562.

UTOGUCHI N, MIZUGUCHI H, SAEKI K, IKEDA K, TSUTSUMI Y, NAKAGAWA S AND MAYUMI T. (1995*a*). Tumor-conditioned medium increases macromolecular permeability of endothelial cell monolayer. *Cancer Lett.*, **89**, 7–14.

UTOGUCHI N, IKEDA K, SAEKI K, OKA N, MIZUGUCHI H, KUBO K, NAKAGAWA S AND MAYUMI T. (1995*b*). Ascorbic acid stimulates barrier function of cultured endothelial cell monolayer. *J. Cell Physiol.*, **163**, 393–399.

WARD JD, HADFIELD MG, BECKER DP AND LOVINGS ET. (1974). Endothelial fenestrations and other vascular alterations in primary melanoma of the central nervous system. *Cancer*, **34**, 1982–1991.

WHEALEY EM, VINCENT PA, MCKEOWN-LONGO PJ AND SABA TM. (1993). Effect of fibronectin on permeability of normal and TNF-treated lung endothelial monolayers. *Am. J. Physiol.*, **264**, R90–R96.